# Draft Genome Sequence of *Stenotrophomonas maltophilia* Strain UV74 Reveals Extensive Variability within Its Genomic Group

**DOI:** 10.1128/genomeA.00611-15

**Published:** 2015-06-11

**Authors:** Oscar Conchillo-Solé, Daniel Yero, Xavier Coves, Pol Huedo, Sònia Martínez-Servat, Xavier Daura, Isidre Gibert

**Affiliations:** a Institut de Biotecnologia i de Biomedicina (IBB), Universitat Autònoma de Barcelona (UAB), Bellaterra (Cerdanyola del Vallès), Barcelona, Spain; b Departament de Genètica i de Microbiologia, Universitat Autònoma de Barcelona (UAB), Bellaterra (Cerdanyola del Vallès), Barcelona, Spain; c Catalan Institution for Research and Advanced Studies (ICREA), Barcelona, Spain

## Abstract

We report the draft genome sequence of *Stenotrophomonas maltophilia* UV74, isolated from a vascular ulcer. This draft genome sequence shall contribute to the understanding of the evolution and pathogenicity of this species, particularly regarding isolates of clinical origin.

## GENOME ANNOUNCEMENT

Although *Stenotrophomonas maltophilia* is an uncommon agent in human bacterial diseases, it can lead to serious nosocomial infections in immunocompromised patients (1, 2). The high genetic diversity exhibited by *S. maltophilia* clinical isolates represents a major limitation for the study of its epidemiology (3, 4). A more alarming characteristic is that most isolates are found to have intrinsic or acquired resistance mechanisms to a large number of antibiotic classes (5). Moreover, the formation of biofilms through quorum-sensing (QS) signals reduces antimicrobial effectiveness (5). *S. maltophilia* strain UV74 was isolated from a vascular ulcer in the Hospital Municipal de Badalona (Barcelona, Spain) during 2009. UV74 is a multidrug-resistant (MDR) organism, showing resistance to amikacin, imipenem, tetracycline, kanamycin, ciprofloxacin, and norfloxacin (6). This isolate also showed significantly high virulence in a zebrafish infection model and formed biofilm and adhered to HeLa cells (6). In this isolate, the diffusible signal factor (DSF)-mediated QS system is regulated by a new *rpf* cluster variant (7).

Genomic DNA was extracted with a GenElute bacterial genomics DNA kit (Sigma-Aldrich). Two different DNA fragment libraries were created and whole-genome sequencing was performed using Illumina MySeq technology at the genomics core facility of Universitat Autònoma de Barcelona, obtaining two different samples of reads. Paired-end reads were scanned for adapters using the NCBI UniVec library and the UCSC blat program (8). Detected adapters were selected and removed with Cutadapt (9) and Skewer (10) and the remaining reads were trimmed to different sizes according to the quality results obtained with FastQC. All possible combinations of trimmed reads from both samples were assembled *de novo* with VelvetOptimiser 2.2.5 (11), relying on Velvet 1.2.0 (12). Contig reorder and improvement was performed with the programs ABACAS and IMAGE, respectively, from the PAGIT package version 1 (13). As a last step, contigs were reordered once again with Mauve (14), using the *S. maltophilia* D457 complete genome (15) as the template. The final assembly resulted in 179 contigs (GC content of 66.65%) with an *N*_50_ contig size of 44,960 nucleotides and covering a total of 4,889,583 bp. The average length of the contigs is 27.3 kb, and the largest contig is 283,791 bp long.

Genome annotation was performed by the NCBI Prokaryotic Genome Annotation Pipeline version 2.10 (rev. 463717), and 4,546 genes were predicted, of which there are 4,382 coding gene sequences (CDS), 89 pseudogenes, 6 rRNAs (5S, 16S, and 23S), 68 tRNAs, and 1 noncoding RNA (ncRNA). Previous multilocus sequence typing (MLST) analysis revealed that UV74 belongs to a new sequence type (http://pubmlst.org/smaltophilia/), being genetically very similar to the model MDR strain D457 (15) and the clinical isolate M30 (16), all three clustering in the same genomic group. Among the predicted CDS of UV74, 3,653 (83.4%) are shared by all three strains, with 299 CDS (6.8%) being exclusive to UV74. Despite the genetic similarity between the three strains in this genomic group, most of their core proteins (77%) showed some degree of variability. Consequently, this new genome may contribute to our understanding of how these bacteria evolve to adapt to different environments.

### Nucleotide sequence accession numbers. 

This whole-genome shotgun project has been deposited at DDBJ/EMBL/GenBank under the accession number LBFT00000000. The version described in this paper is version LBFT01000000.
